# LncRNA and mRNA profiling during activation of tilapia macrophages by HSP70 and *Streptococcus agalactiae* antigen

**DOI:** 10.18632/oncotarget.21427

**Published:** 2017-09-30

**Authors:** Honglin Luo, Huizan Yang, Yong Lin, Yongde Zhang, Chuanyan Pan, Pengfei Feng, Yanling Yu, Xiaohan Chen

**Affiliations:** ^1^ Guangxi Key Laboratory for Aquatic Genetic Breeding and Healthy Aquaculture, Guangxi Institute of Fishery Sciences, Nanning, P.R. China; ^2^ Guangxi Medical University, Nanning, P.R. China; ^3^ College of Animal Science and Technology, Guangxi University, Nanning, P.R. China

**Keywords:** long non-coding RNA, *oreochromis niloticus*, macrophages, HSP70, *Streptococcus agalactiae*

## Abstract

**Objectives:**

To investigate the lncRNA profiling during tilapia peritoneal macrophages (TPMs) activation and discuss the relationship between lncRNA and mRNA.

**Materials and Methods:**

RNA sequencing was used to investigate the lncRNA and mRNA profiles of TPMs activation following stimulation with *Streptococcus agalactiae* (Sa) antigen, heat shock protein 70 (HSP70) and HSP70+Sa. The expressions of lncRNA and mRNA were confirmed by qPCR. 356 lncRNA, 10173 mRNA and 1782 transcripts of uncertain coding potential (TUCP) were differentially expressed by pairwise comparison. These lncRNAs were shorter in length, fewer in exon number and higher in expression levels as compared with mRNAs. 683 lncRNAs and 4320 mRNAs were co-located, while 316 lncRNAs and 9997 mRNAs were in co-expression networks. Seven mRNAs (ANKRD34A, FMODA, GJA3, CNTN5, BMP10, BAI2 and HS3ST6) were involved in both networks of LNC_00035 and LNC_000466. Differentially expressed genes were involved in signaling pathways, such as “phosphorylation”, “cytokine-cytokine receptor interaction”, “endocytosis” and “MHC protein complex”. LNC_000792, LNC_000215, LNC_000035 and LNC_000310, with *cis* and/or *trans* relationships with mRNAs, were also involved in ceRNA network.

**Conclusions:**

These results might represent the first identified expression profile of lncRNAs and mRNAs in tilapia macrophages activated by HSP70 and Sa.

## INTRODUCTION

*Streptococcus agalactiae* (Sa) is one of the most important pathogens causing severe streptococcicosis for tilapia industry of China. Currently, there are no effective chemicals or vaccines available against this disease for commercial use yet. Identification of the immunological responses between Sa and host immune system is of utmost importance given the economic burden of the disease to the aquatic industry. The activation of mammal macrophage is crucial for its immunological functions, and similar functions have been reported in fish macrophages [1–4]. Previous study showed that recombinant tilapia heat shock protein 70 (HSP70) could promote the immune-related gene expression of tilapia peritoneal macrophages (TPMs) after stimulation with streptococcal antigen and HSP70 [5]. In mammals, extracellular HSP70 can increase expressions of TNFα, IL1β, IL6, and IL12 in monocytes, release of TNFα, IL1β, GMCSF, and IFNγ in macrophages and dendritic cells [6, 7], and production of TNFα and IL1β by activating NFκB through TLR2/4-CD14 signaling pathway [8]. These observations suggest that HSP70 might function as a cytokine. Furthermore, HSP70 is capable of breaking through immune tolerance. For instance, HSP70 blocked the tumor-associated antigen tolerance process, and killed specific tumor cells by cytotoxic CD8^+^T cells [9], and several tumor HSP peptide vaccines made great progresses in breast cancer, melanoma, and pancreatic cancer immunotherapy [10–12]. As such, HSP70 is valuable for vaccine development. In fish, environmental stress and pathogen infection could induce the expression of HSP70 [13–15], indicating that fish HSP70 might have similar functions to mammal HSP70. Chen N *et al*. reported that the TNFα and IL1β mRNA in liver, head kidney, and spleen increased after intraperitoneal injection of HSP70 in Wuchang Bream [16], and levels of TNFα and IL1β in grasp carp head kidney increased and was closely correlated with NFκB signaling [17]. This mechanism might be important in the development of anti-Sa vaccine since TNFα could induce chemotactic response, phagocytosis and nitric oxide production [18, 19], and IL1β could further stimulate the production of other functional cytokines in fish [20]. However, the mechanism by which HSP70 may induce macrophage activation in fish has not been clearly clarified yet.

Long non-coding RNA (lncRNA) is known to play a key role in gene regulation network in mouse and human. For example, lncRNA participated in the activation of CD8^+^T cells [21], CD4^+^T cells and CD11C^+^ dendritic cells [22, 23]. Importantly, lncRNAs also play essential roles on macrophage differentiation. For instance, TCONS_00019715 participated in human macrophage polarization [24], and PU.1-associated lnc-MC mediated human mononuclear macrophage differentiation *via* adsorption of miR-199a-5p to increase expression of PU.1 and ACVR1B [25]. However, no reports have been described the roles of lncRNAs on HSP70-induced fish macrophage activation. Herein, we hypothesized that lncRNA may be important for tilapia HSP70 and Sa induced-macrophage activation. To address our hypothesis, we investigated the expression profiles of TPMs treated with Sa antigen and/ or HSP70, and performed a comprehensive analysis of lncRNAs of Nile tilapia macrophage samples by RNA-seq to ascertain the number and nature of differentially expressed transcripts and to determine which biological processes and pathways significantly changed.

## RESULTS

### Identification and features of lncRNAs, TUCPs, and mRNAs in activated TPMs

**Figure 1:**
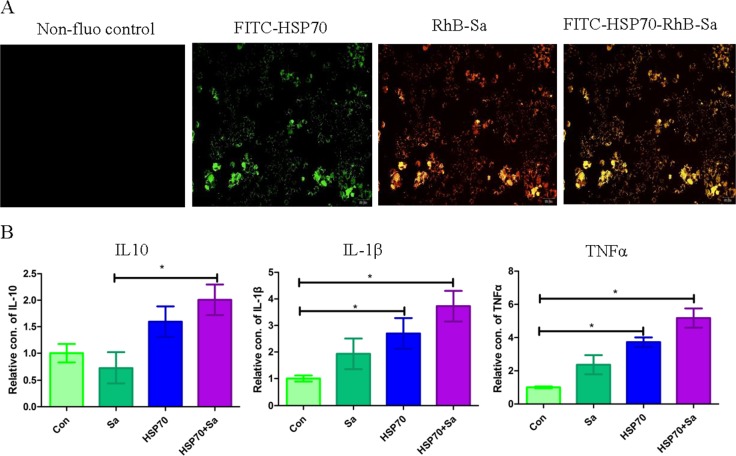
Identification of the interaction of TPMs with HSP70/Sa antigen **(A)** Confirmation of the interaction of HSP70, Sa and HSP70+Sa with TPMs by fluorescence of FITC and RhB. **(B)** QPCR examination of three genes that indicating the activation of TPMs after the stimulation of HSP70, Sa and HSP70+Sa.

**Figure 2:**
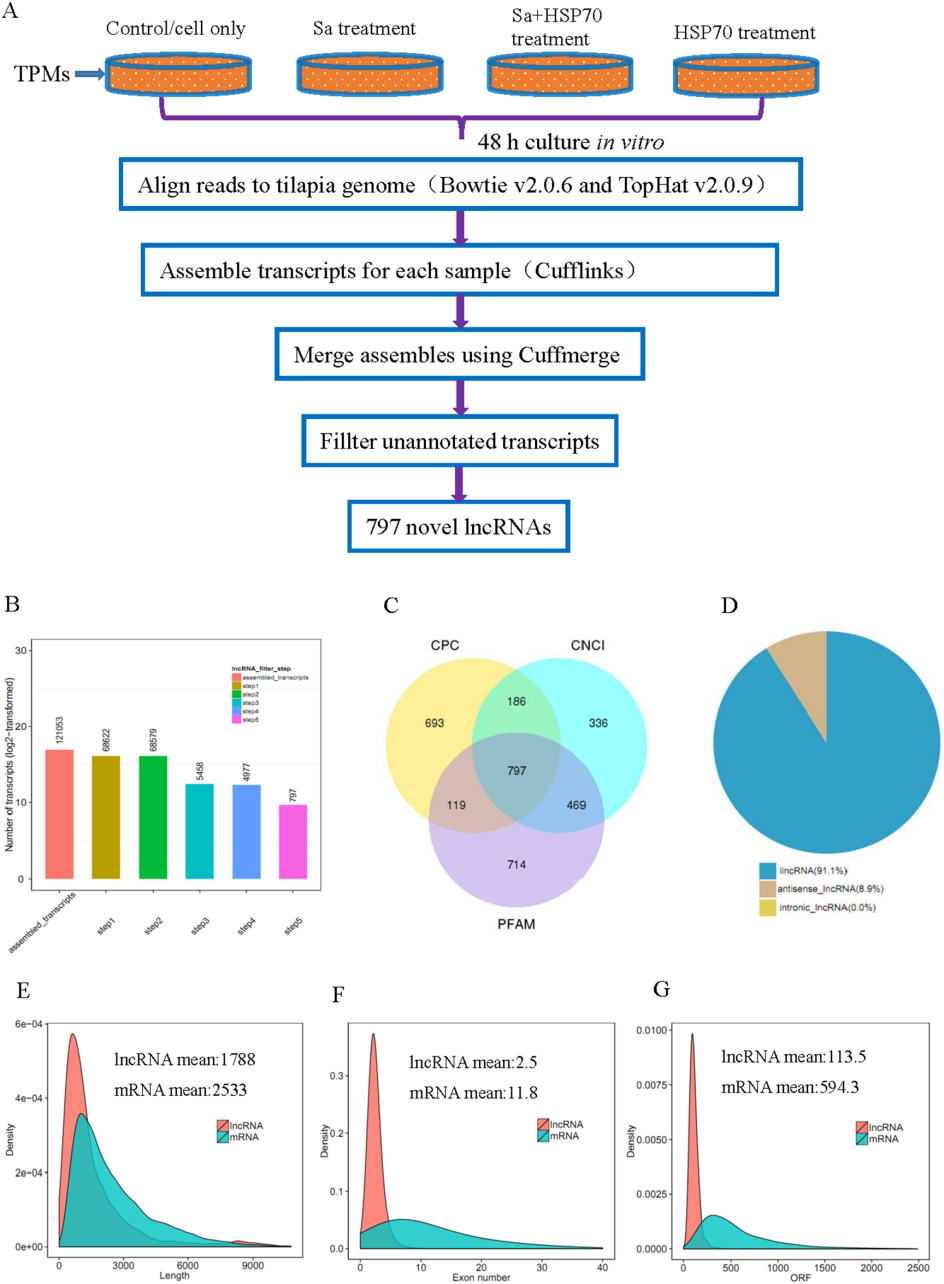
LncRNA filtering **(A)** Experimental workflow and analysis for analyzing RNA-seq data from tilapia peritoneal macrophages (TPMs) treated with *Streptococcus agalactiae* antigen (Sa) and Heat shock protein 70 (HSP70), respectively. **(B)** Numbers of lncRNAs after 5 steps analysis. **(C)** Filtering of lncRNAs by using CPC, CNCI and PFAM softwares. **(D)** Classification of lncRNAs obtained by RNA-seq. **(E)** Length distribution of 26763 coding transtripts and 797 new predicted lncRNAs. **(F)** Exon number distribution of coding transtripts and lncRNAs. **(G)** ORF length distribution of coding transtripts and lncRNAs.

Before RNA sequencing, the interaction of HSP70, Sa and HSP70+Sa with TPMs was confirmed by using fluorescein labelling and qPCR examination. As shown in Figure 1, fluorescein-labelled HSP70, Sa and HSP70+Sa were intensively internalized by TPMs and the expression of three genes, IL10, IL1-β and TNFα, the indicator of the activation of TPMs, was significantly increased after stimulation with HSP70 and HSP70+Sa compared to the control group. We then analyzed the RNA-Seq data from these samples, of which 83 to 122 million raw reads and 80 to 111 million clear reads per sample were obtained. A total of 121053 transcripts were assembled by Cufflinks (Figure 2B). Three tools, Coding Potential Calculator (CPC), Pfam-scan (PFAM) and Coding-Non-Coding-Index (CNCI) were used to remove potential coding transcripts, and finally 797 putative non-coding transcripts were retained as lncRNAs (Figure 2C). Among them, 91.1% were intergenic lncRNAs (lincRNA) and 8.9% were antisense lncRNAs (Figure 2D). The predicted lncRNAs were shorter in length than protein coding transcripts (Figure 2E), and their putative genes contained fewer exons (Figure 2F). We found that lncRNAs in activated TPMs were shorter in length than lncRNAs in zebrafish (1113 nt on average), human (1000 nt on average), and mouse (550 nt on average), and contained fewer exons than zebrafish (2.8 exons on average), human (2.9 exons on average) and mouse (3.7 exons on average). Furthermore, the identified lncRNAs were generally shorter in open reading frame (ORF) length than protein coding genes (Figure 2E, 2F, and 2G). In addition, we found 26763 mRNAs and 4180 transcripts of uncertain coding potential (TUCP).

**Figure 3:**
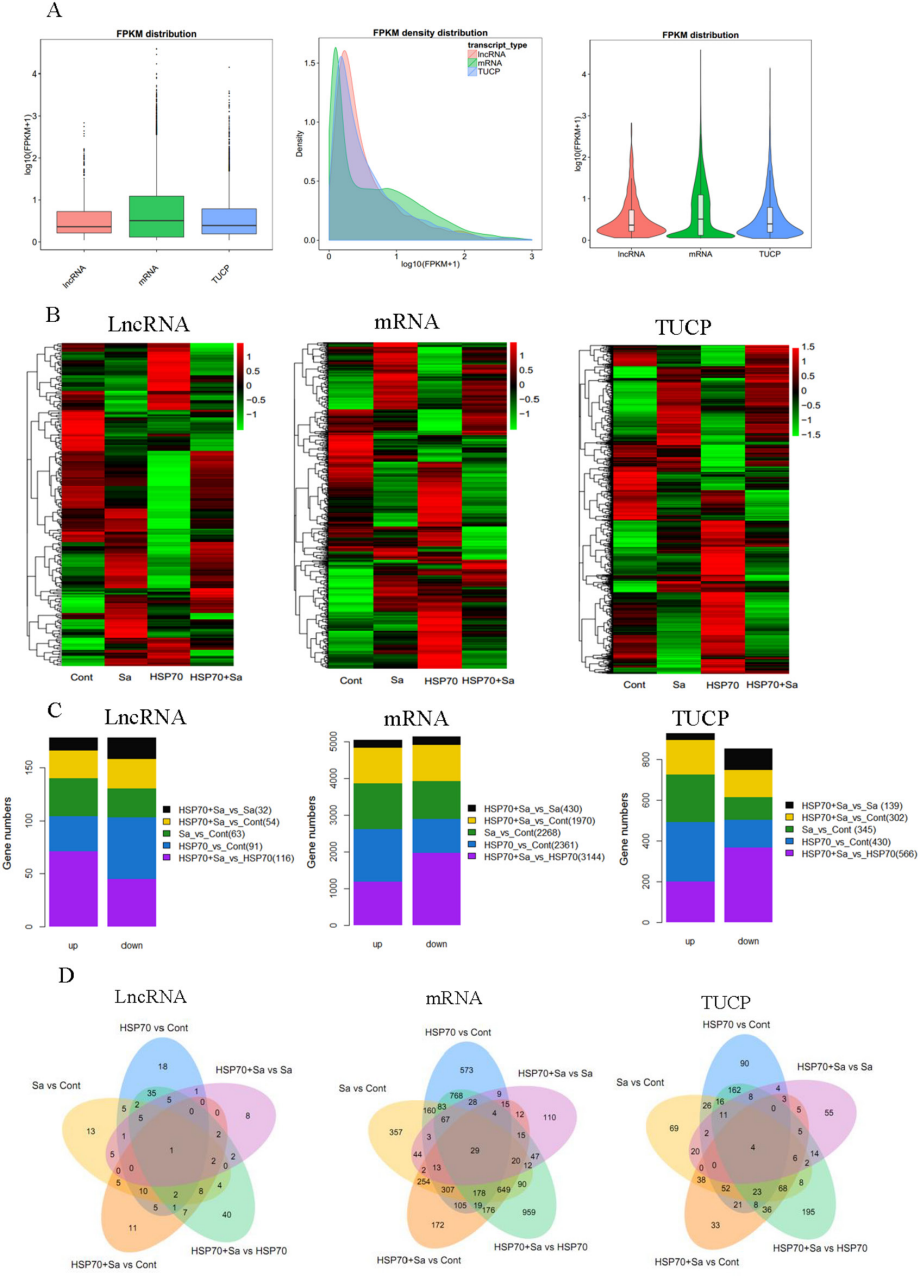
Gene quantification, expression profiling and number of differentially expressed genes **(A)** Quantification of transcripts obtained from tilapia peritoneal macrophages (TPMs) treated with Sa and HSP70, respectively. **(B)** Heatmaps of the transformed expression values for transcripts (lncRNA, mRNA and TUCP). **(C)** Statistics of differentially expressed lncRNA, mRNA and TUCP in five comparison groups (HSP70 vs Cont, Sa vs Cont, HSP70+Sa vs Cont, HSP70+Sa vs HSP70 and HSP70+Sa vs Sa). **(D)** Venn diagram of differentially expression genes in five comparison groups.

By pairwise comparison, we found lncRNA, mRNA and TUCP were differentially distributed among groups. Totally, 356 lncRNAs, 10173 mRNAs and 1782 TUCPs were differentially expressed genes from pairwise comparisons (Figure 3A, 3B, Supplementary Tables 1, 1-1, 1-2, 1-3). The most increased lncRNAs (71) were found in HSP70+Sa vs. HSP70 comparison, while the most decreased lncRNAs were among HSP70+Sa vs. Sa (20). For mRNAs, HSP70+Sa vs. Control had the most increased genes (1426), while HSP70+Sa vs. Sa had the lowest number of increased genes (217). For TUCP, the most increased and decreased genes were found in the same comparisons of mRNAs (293 and 106, respectively) (Figure 3C). As for lncRNAs, 18 in HSP70 vs. Control, 8 in HSP70+Sa vs. Sa, 40 in HSP70+Sa vs. HSP70, 11 in HSP70+Sa vs. Sa and 13 in Sa vs. Control were found specifically expressed in each comparison, while only LNC_000014 was commonly expressed in all groups (Figure 3D), and its fragments per kilobase of exon per million fragments mapped (FPKM) distribution in Control group was significantly higher than Sa, HSP70 and HSP70+Sa groups, respectively (data not shown). None of these lncRNAs have been previously identified. For mRNAs, 573, 110, 950, 172 and 357 were specifically expressed in HSP70 vs. Control, HSP70+Sa vs. Sa, HSP70+Sa vs. HSP70, HSP70+Sa vs. Sa and Sa vs. Control comparison, respectively (Supplementary Table 1-2). Furthermore, 29 mRNAs were commonly expressed in all groups. A number of TUCPs were expressed in each comparison, but only four were commonly expressed in all comparisons (Figure 3D, Supplementary Table 1-3).

### Validation of lncRNAs and mRNAs

**Figure 4:**
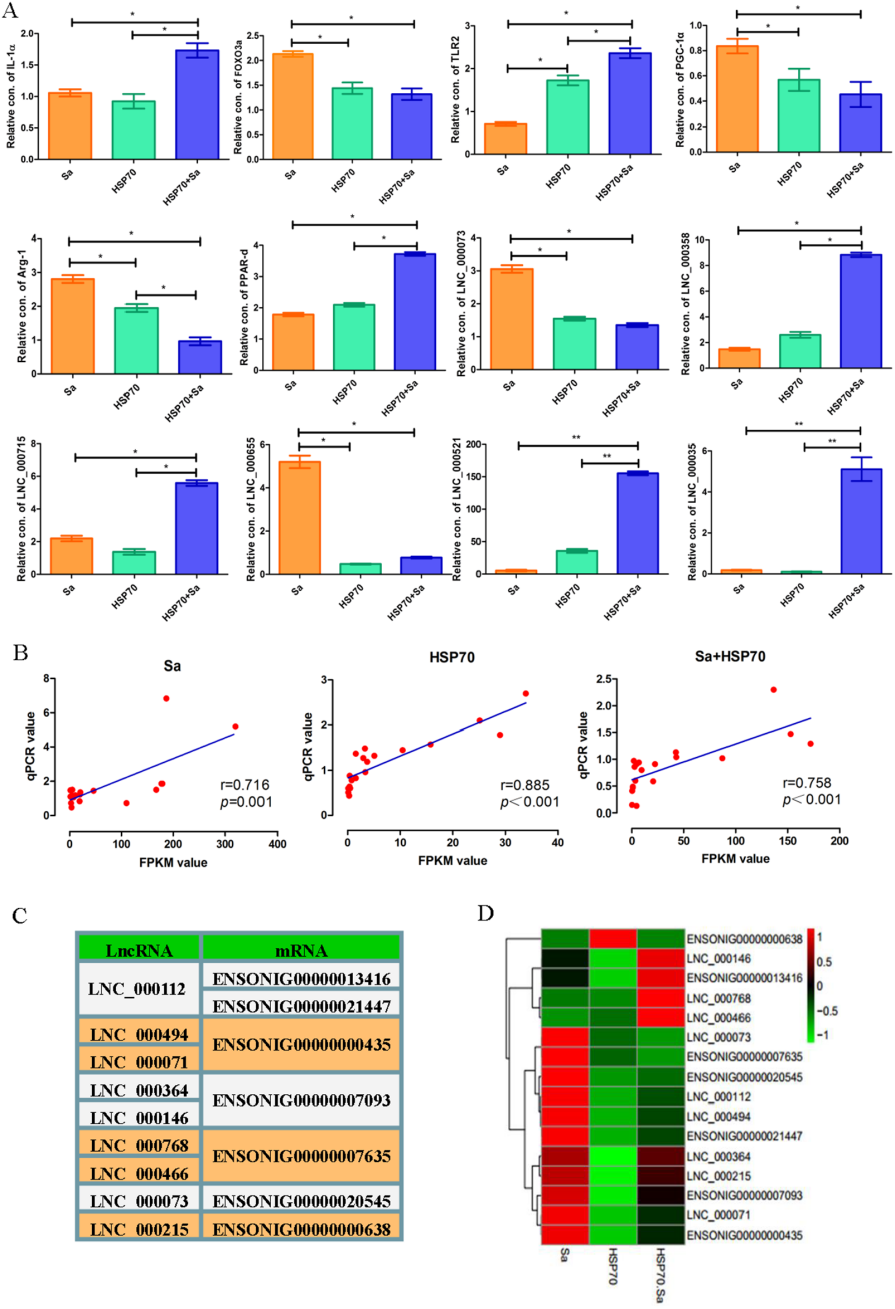
QPCR validation of candidate lncRNAs and mRNAs obtained from tilapia peritoneal macrophages (TPMs) treated with Sa and HSP70, respectively **(A)** The relative expression levels of 27 candidate lncRNAs and 34 mRNAs were detected by qPCR in TPMs treated with Sa and HSP70. The 2 ΔCt values of the lncRNAs and mRNAs were determined by subtracting the β-actin ΔCt value. Twelve lncRNAs and mRNAs were presented to represent the results of qPCR validation. ^*^ indicates p< 0.05. **(B)** Nineteen lncRNAs and mRNAs in three groups were selected randomly to evaluate the relationship of microarray results and qPCR value which was analyzed by Spearman's coefcients. **(C)** Selected lncRNAs and mRNAs having trans or cis relationship. Some of lncRNA or mRNA has more than one corresponding mRNA or lncRNA. **(D)** Heatmap of the selected lncRNAs and mRNAs based on the QPCR results.

To validate the RNA-seq results, 27 candidate lncRNAs and 34 mRNAs were detected by quantitative polymerase chain reaction (qPCR) in TPMs treated with Sa and/or HSP70 (primers listed in Supplementary Table 2). Twelve lncRNAs and mRNAs (IL-12α, FOXO3a, TLR2, PGC-1α, Arg-1, PPAR-d, LNC_000073, LNC_000358, LNC_000715, LNC_000655, LNC_000521 and LNC_000035) were represented to the results of qPCR validation (Figure 4A). The selected lncRNAs and mRNAs were significantly differentially expressed at least in one comparison group, and their associated genes have previously been reported to be involved in TPMs activation. For instance, IL12α, TLR2, PGC-1α and Arg-1 have been documented in macrophage activation in mouse and human [6–9]. Spearman's coefficient was used to evaluate the relationship of microarray results (FPKM value) and qPCR analyses, and the expression patterns of these genes were in agreement with the RNA-seq findings (Figure 4B). Next, we randomly selected 7 mRNAs from the 34 mRNAs and analyzed their relationships with their targeted lncRNAs, and found that three of the selected mRNAs corresponded to two lncRNAs, while two mRNAs corresponded to one lncRNA, with *cis* (Supplementary Table 3) or *trans* (Supplementary Table 4) relationship. The other mRNAs had one to one correspondence with lncRNA (Figure 4C). Subsequently, the expression levels of these lncRNAs and their corresponding mRNAs were analyzed (Figure 4D), and two types of expression profiles between lncRNAs and mRNAs (*cis* and *trans*) were demonstrated, which was consistent with the FPKM predictions (Figure 4C, 4D). For example, ENSONIG034160R113416 and ENSONIG034160R121447 corresponded to LNC_000112, which was highly expressed in Sa group and lowly expressed in HSP70 and HSP70+Sa group. Similar expression profile was found in ENSONIG034160R121447, while the opposite expression profile was found in ENSONIG034160R113416.

### Correlation of expression patterns between pairs of target genes

**Figure 5:**
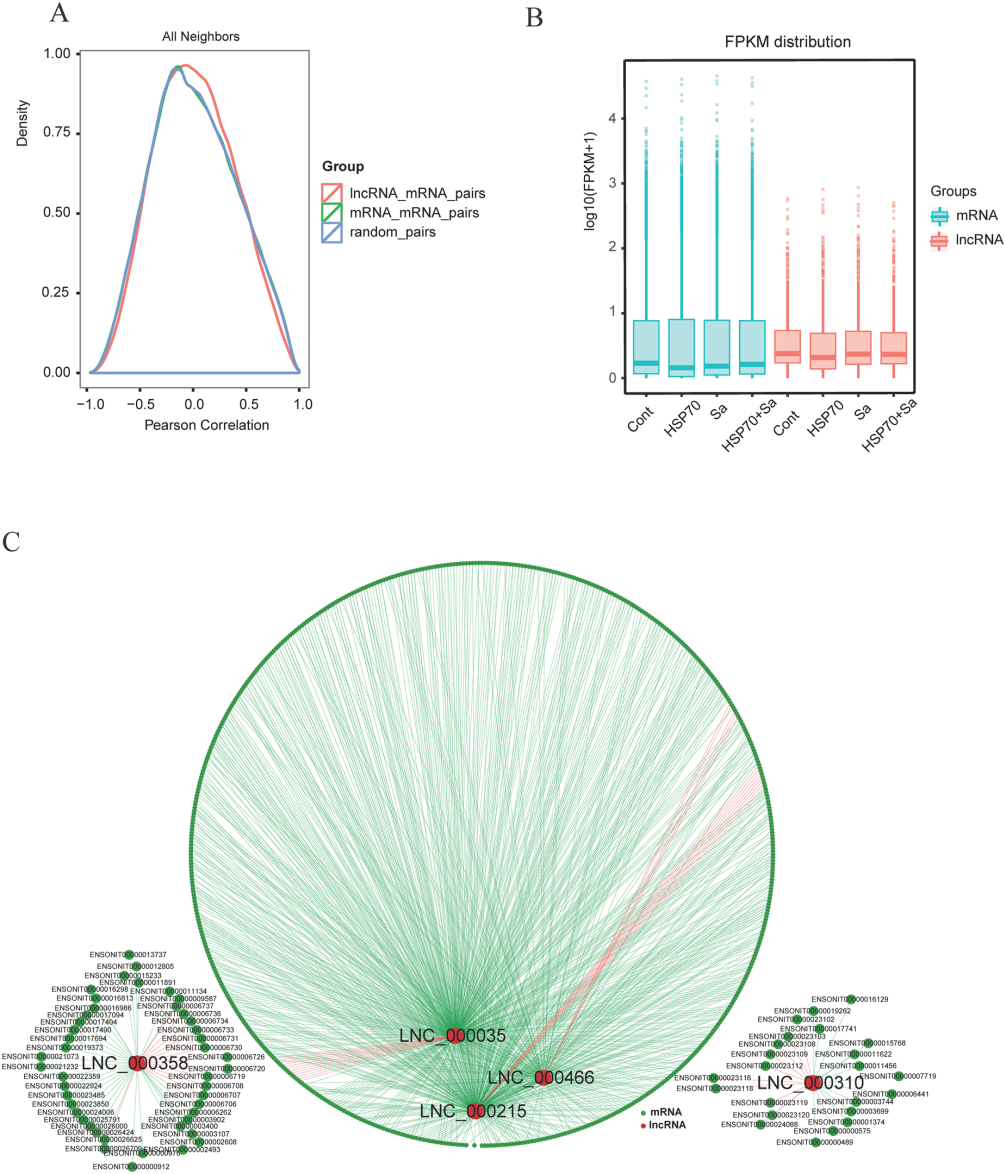
Correlation of expression patterns between pairs of target genes **(A)** Shown are distributions of Pearson correlation coefficients in expression levels between either 400000 pairs of coding gene neighbors (green), 100000 pairs of lncRNAs (red) and 800000random pairs of genes (blue). **(B)** Box plots showing the expression feature of lncRNA and mRNA in each samples. **(C)** Shown are networks of five selected lncRNAs that associated with both cis and trans relationship with mRNAs. Red line represents cis relationship, green line represents trans relationship. Red circle represents lncRNAs and dark green circle represents mRNAs.

To investigate the relationship between lncRNAs and their neighboring coding genes, we randomly selected 400000 mRNAs, 100000 lncRNAs, and 800000 random gene pairs formed by lncRNAs and their neighboring genes. We observed a more correlated expression pattern of lncRNAs with their neighboring gene pairs (mean correlation: 0.01899) than random coding gene pairs (mean correlation: 0.01687), and the expression pattern of lncRNAs showed a relatively higher correlation than coding gene pairs (mean correlation: 0.01752) (Figure 5A). We also found that lncRNAs were generally expressed at higher levels than mRNAs (Figure 5B). In addition, we observed that 683 lncRNAs and 4320 mRNAs were co-located (namely, *cis*), while 316 lncRNAs and 9997 mRNAs were in co-expression (namely, *trans*) network. Next we randomly selected five lncRNAs, LNC_00035, LNC_000215, LNC_000310, LNC_000358 and LNC_000466, to demonstrate the relationships between lncRNAs and corresponding mRNAs. As shown in Figure 5C, the five lncRNAs had both *cis* and *trans* relationships with their corresponding mRNAs. For example, 371 mRNAs were involved in the *trans* network with LNC_00035, which also had 11 co-located mRNAs. Specifically, we found seven mRNAs (ANKRD34A, FMODA, GJA3, CNTN5, BMP10, BAI2, and HS3ST6) were involved in both networks of LNC_00035 and LNC_000466. However, some lncRNAs had only *cis* or *trans* relationships with mRNAs (data not shown).

### Differential expression cluster analysis and functional prediction of lncRNAs and mRNAs in activated TPMs

**Figure 6:**
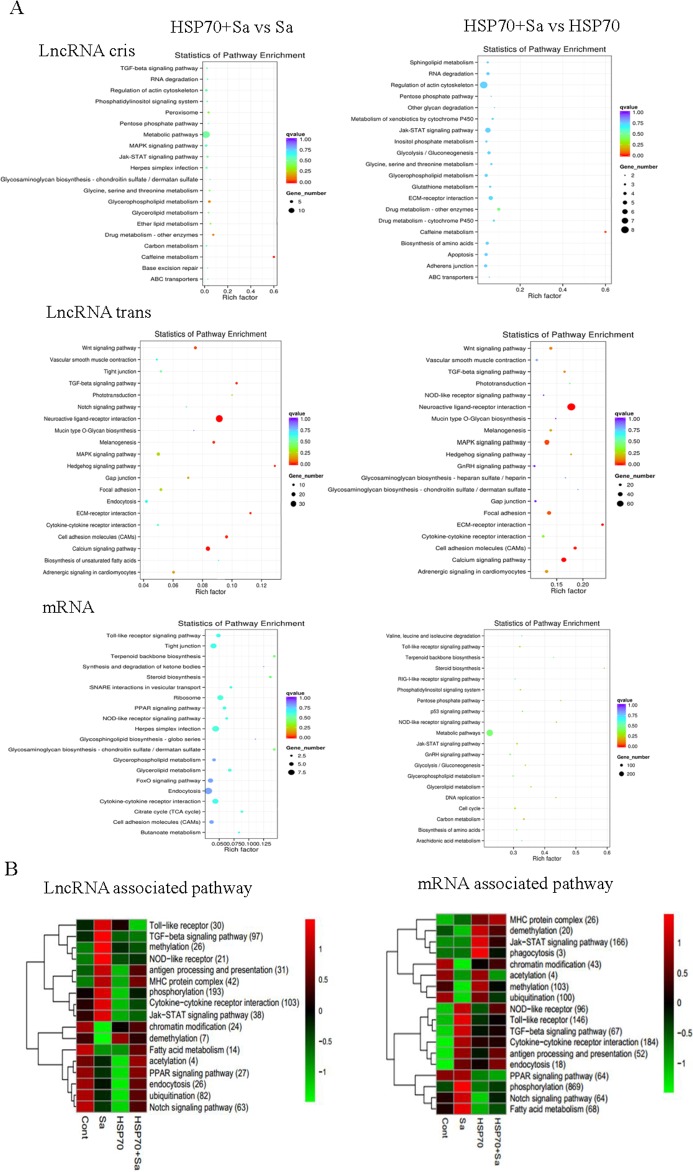
KEGG pathways involved in process of tilapia peritoneal macrophages (TPMs) stimulated with Sa and HSP70, respectively **(A)** Pathways involved in two important comparison groups (HSP70+Sa vs Sa, HSP70+Sa vs HSP70) in lncRNAs and mRNAs category. **(B)** KEGG annotation of different expression lncRNAs and mRNAs that might be involved in macrophage activation process. The number in each annotation represents total counts of genes that might be involved in the pathway in all four comparison groups.

To further predict the function of lncRNAs in activated TPMs, we performed a gene ontology (GO) analysis with the selected mRNAs which neighbor lncRNAs or have high co-expression with lncRNAs in four comparison groups. GO terms enrichment was found mostly in mRNAs *trans* with lncRNAs but not the mRNAs *cis* with lncRNAs. The highest number of differentially expressed genes (*p*-value < 0.05) in biological process were related to “regulation of biological process” in HSP70+Sa vs. HSP70 group, “nucleic acid metabolic process” in HSP70+Sa vs. Sa and Sa vs. Control groups, and “biological regulation” in HSP70 vs. Control group (Supplementary Figure 1). Kyoto Encyclopedia of Genes and Genomes (KEGG) analysis revealed that both lncRNAs with *cis* and *trans* relationships with mRNAs had enriched pathways. For example, metabolic pathways were the most enriched pathways in all comparison groups in either *cis* or *trans* relationship (Figure 6A, Supplementary Figure 2). While the “regulation of actin cytoskeleton” and “Jak-STAT signaling pathway” were the most enriched pathways in HSP70+Sa vs. HSP70 group in neighbor lncRNAs, no significant enrichment pathways were found in *trans* lncRNAs in this group (Figure 6A). As for differentially expressed mRNAs, the most significantly enriched pathway was “endocytosis”, which was mainly in HSP70+Sa vs. Sa and Sa vs. Control groups. Metabolic pathways still found to be enriched in HSP70+Sa vs. HSP70 and HSP70+Sa vs. Control groups. We also found that “tight junction”, “ribosome”, “Herpes simples infection”, “glycerophospholipid metabolism”, “FOXO signaling pathway”, “cytokine-cytokine receptor interaction”, “cell adhesion molecules”, “Toll-like receptor signaling pathway” and “cell cycle” pathways were dominated during TPMs activation (Figure 6A, Supplementary Figure 2). We next determined which pathways were the most enriched during TPMs activation, and found that “phosphorylation”, “cytokine-cytokine receptor interaction”, “TGF-beta signaling pathway”, “ubiquitination” and “notch signaling pathway” were the top five pathways co-related to lncRNAs, although enrichment level in four comparison groups was divergent (Figure 6B). Compared to lncRNAs, the top five most enriched pathways in mRNAs were “phosphorylation”, “cytokine-cytokine receptor interaction”, “Jak-STAT signaling pathway”, “Toll-like receptor” and “methylation”. The concerned pathways during TPMs activation, “antigen processing and presentation”, “endocytosis” and “MHC protein complex” were also found to be enriched in both lncRNA and mRNA categories (Figure 6B).

### Construction of competing endogenous RNAs (ceRNA) network

**Figure 7:**
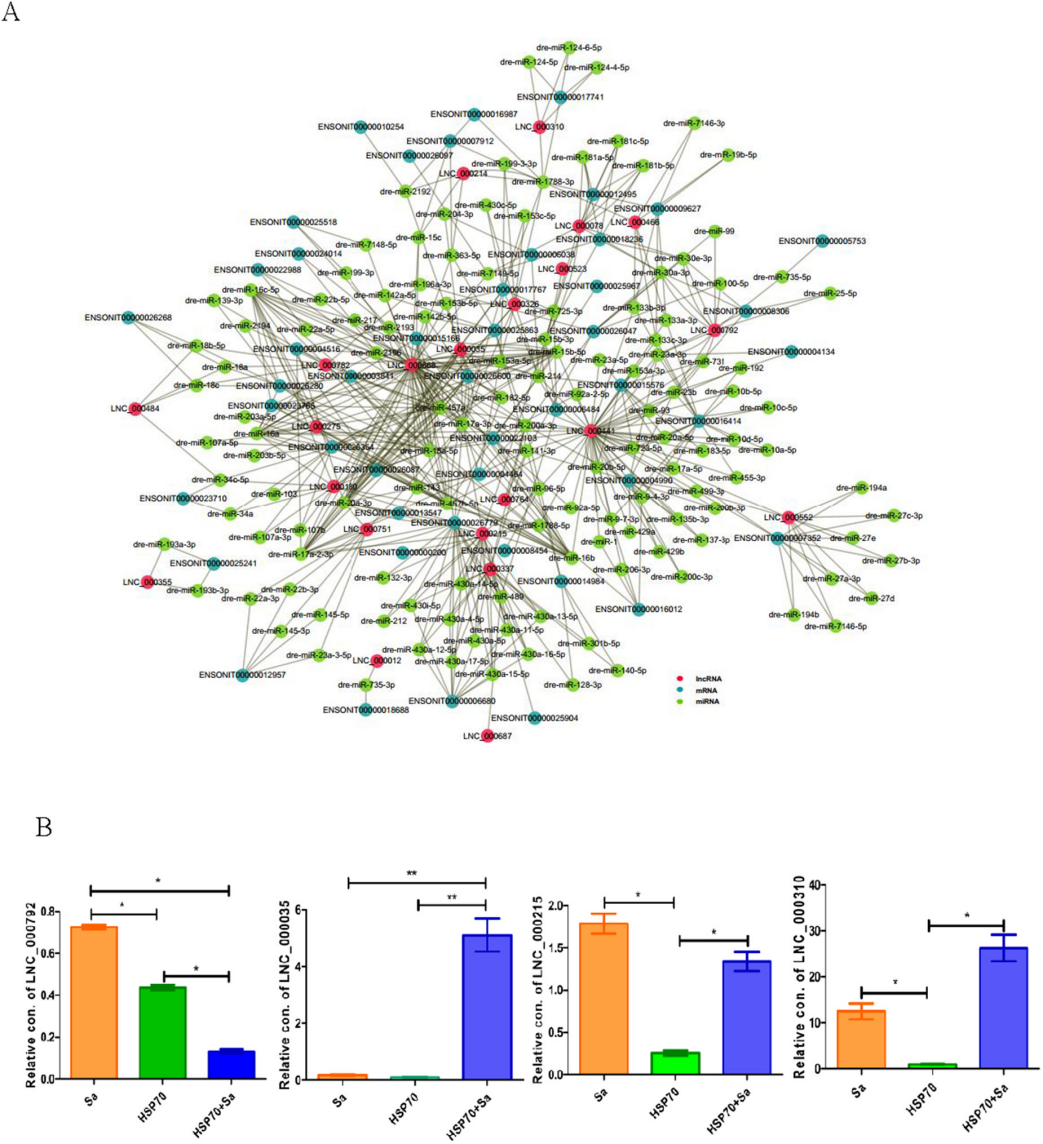
ceRNA networks and four lncRNAs involved both in cis and trans relationships **(A)** The ceRNA network built from TPMs treated with Sa and HSP70, respectively. This ceRNA network including 130 miRNAs, 22 lncRNAs and 53 mRNAs that interact each other. Red circle represents lncRNAs, dark green circle represents mRNAs and green circle represents miRNAs that involved in ceRNA network. **(B)** QPCR identification of four lncRNA, LNC_000792, LNC_000215, LNC_000035 and LNC_000310 that found in the relationships of cis and trans of lncRNAs, and also involved in ceRNA network. The 2 ΔCt values of the lncRNAs were determined by subtracting the β-actin ΔCt value. ^*^ indicates p< 0.05.

Since mRNAs and lncRNAs can interact with miRNAs through their miRNA recognition elements (MREs) within a ceRNA network, we scanned all differentially expressed lncRNAs and mRNAs during TPMs activation against fish miRNA database (Supplementary Table 5). We found that a total of 22 lncRNAs, 53 mRNAs and 126 miRNAs were involved in a ceRNA network. As shown in Figure 7A, LNC_000668, LNC_000441, LNC_000215, LNC_000337 and LNC_000782 were the top five lncRNAs with mostly interacted miRNAs and mRNAs. For instance, 13 mRNAs and 24 miRNAs were involved in the ceRNA network of LNC_000668. However, LNC_000523, LNC_000687 and LNC_000012 had only one miRNA and one mRNA interaction. We also found that LNC_000792 (4 mRNAs and 8 miRNAs), LNC_000215 (3 mRNAs and 18 miRNAs), LNC_000035 (5 mRNAs and 8 miRNAs) and LNC_000310 (1 mRNAs and 4 miRNAs), with *cis* and/or *trans* relationships with mRNAs in lncRNA targets data, were also involved in ceRNA network. Subsequent investigation showed that the expressions of these lncRNAs were divergent. For instance, LNC_000792 was significantly increased in Sa- and HSP70-stimulated groups compared to HSP70+Sa group, while LNC_000035 had an opposite expression profile in these groups (Figure 7B).

## DISCUSSION

Tilapia HSP70 could activate TPMs [5], which was consistent with previous reports on macrophage activation in mammals [6–8]. However, the underlying mechanisms involved during this process remain unknown. LncRNA plays crucial roles in various biological and immunological processes [21–23, 26, 27]. Nonetheless, the roles of lncRNAs during HSP70-induced macrophage activation in fish have yet to be elucidated. In examining the role of lncRNAs in TPMs activation by HSP70 and/or Sa, we identified 797 novel lncRNAs, 26763 mRNAs and 4180 TUCP, among which 356 lncRNA, 10173 mRNA and 1782 TUCP were differentially expressed by pairwise comparison. To the best of our knowledge, this is the first report on lncRNA expression profile during HSP70-associated macrophage activation in fish, providing clues on immune regulation of macrophage activation in other fish and potentially mammals.

The aim of this study was to identify lncRNA during TPMs activation. We found that 797 transcripts were predicted as novel lncRNAs in tilapia. Previous studies showed that lncRNAs could be classified into six types: sense, anti-sense, bidirectional, intronic, intergenic and small RNA (sRNA) host lncRNA [28]. In our study, only two types of lncRNAs (intergenic and anti-sense) were found. This might be attributed to the lack of tilapia lncRNA database currently, and thus some lncRNAs could not be filtered and identified. This, to some extent, was supported by the fact that more TUCPs (4180) than lncRNAs (797) were identified in our study. By pairwise comparison, we found lncRNAs and mRNAs were differentially distributed among groups. For lncRNAs, the most increased lncRNAs were found in HSP70+Sa vs. HSP70 comparison, while the most decreased lncRNAs were among HSP70+Sa vs. Sa comparison. For mRNAs, HSP70+Sa vs. Control had the most increased genes, while HSP70+Sa vs. Sa had the lowest increased genes. These data indicated that the most increase and decrease lncRNAs mainly involved in HSP70-associated TPMs activation. This, to some extent, was consistent with our previous report and others that HSP70 could help to activate macrophages [5–8], and this was verified by qPCR results in our study from both mRNA and lncRNAs expression levels.

Recent studies reported that some lncRNAs regulate gene expression of their neighborhood in *cis* [29], and non-neighbor genes in *trans* [30]. In the current study, we found that 683 lncRNAs with 4320 mRNAs were in *cis*, while 316 lncRNAs with 9997 mRNAs were in *trans*, indicating both *cis* and *trans* regulatory models dominate and may play essential roles on TPMs activation. Take LNC_000310 as an example, 14 mRNAs were involved in *trans* and 9 mRNAs were in *cis*, indicating LNC_000310 was involved in both *trans* and *cis* regulations. Evidence suggests that lncRNAs may regulate and associate with expression of neighboring mRNAs [27]. Furthermore, we found that among the 9 mRNAs in *cis* relationship of LNC_000310, IL13Ra1 chain is crucial for induction of the alternative macrophage activation by IL13 [31], and can be regulated by miR155 in establishment of an M2 phenotype in human macrophages [32]. Additionally, TIRAP, in *cis* with LNC_000358, was reported to be essential in activation of TLR2/4 shared signaling [33], which is important in macrophage activation [34]. Similarly, mRNAs in *trans* with lncRNAs may also be involved in the activation of TPMs. For instance, we found that 250 mRNAs were involved in *trans* network of LNC_000215. Among them, at least GFRA3 [35, 36], NFATC1 [37] and ADCYAP1a [38, 39] might be involved in the activation processes of macrophages. These suggest that some lncRNAs, both in *cis* and/or *trans* with mRNAs, may exert their functions through their associated mRNAs, which play crucial roles in HSP70-induced TPMs. However, these predicted functions of lncRNAs need experimental verification in our future studies.

Our GO analysis revealed that “metabolic process” and “biological regulation” were important processes during TPMs activation, and the KEGG pathway analysis supported these findings. In mammals, metabolic pathways have been documented to be involved in macrophage activation [40–42]. Generally, classic macrophage activation (M1) is involved in the fast anaerobic glycolysis, while alternatively activated macrophages (M2) rely on aerobic respiration, which requires pyruvate feeding into the TCA cycle and oxidative phosphorylation to produce more efficient but slower ATP than glycolysis alone [43]. For instance, LPS-induced M1 activation could induce metabolic changes leading to increased glycolysis and reduced oxidative phosphorylation, while IL4/IL13-induced M2 activation could increase oxidative phosphorylation [44, 45]. In our study, although many other pathways were enriched, we observed that “metabolic pathway” was involved in almost all compared groups in both lncRNA and mRNA categories, suggesting that this pathway might be very important during TPMs activation. We then found that in the metabolic pathway, GPIb (glucose-6-phosphate isomerase b), DAO.2 (D-amino-acid oxidase, tandem duplicate 2), GATC (glutamyl-tRNA amidotransferase subunit C), PLPP1a (phospholipid phosphatase 1a), ENSONIG034160R102868, ENSONIG034160R120602 and ENSONIG034160R120603 were enriched and varied in almost all compared groups. These genes are associated with glycolysis and phosphorylation, indicating they might be involved in the metabolic reprograming in TPMs activation similar to that in mammals. We also found that regulatory pathway containing “phosphorylation” was the most enriched number pathway involved in both lncRNA and mRNA categories, highlighting its regulatory roles during TPMs activation. Previous reports showed that phosphorylation was associated with glycolysis during macrophage polarization [42, 46, 47]. This allows the speculation establish that glucose metabolic pathway and phosphorylation pathway might be connected and quite important in TPMs similar to mammals. However, this possibility needs experimental verification.

The ceRNA hypothesis was proposed as a novel regulatory mechanism between non-coding RNA and coding RNA [48, 49]. The ceRNA genes are mediated by the miRNAs interacting with lncRNAs through miRNA-binding sites, thereby regulating gene expression [50]. The crosstalk of ceRNA network has been documented in various diseases [51] and biological processes [25], but few studies have been reported in macrophage activation even in mammals. In this study, we constructed lncRNA-miRNA-mRNA ceRNA network during HSP70-associated TPMs activation, providing crucial hints for detection of the key RNAs of ceRNA-mediated gene regulatory network. We speculate that this might be the first reported ceRNA network in fish macrophage. In this network, we found that 22 lncRNAs, 53 mRNAs and 126 miRNAs, among which the LNC_000668 (23 miRNAs, 13 mRNAs), LNC_000441 (44 miRNAs, 5 mRNAs), LNC_000215 (18 miRNAs, 3 mRNAs), LNC_000337 (15 miRNAs, 2mRNAs) and LNC_000782 (13 miRNAs, 2 mRNAs) were the top five lncRNAs with mostly interacted miRNAs and mRNAs. Although all the lncRNAs and most of the mRNAs in the network were uncharacterized, we still found that dre-miR-457a, which can accelerate the proliferation of B cells by regulating the expression of genes controlling cell cycle in mammals [52], might interact with LNC_000668 to regulate expression of XK-related protein (ENSONIT034160R115166), which mediates phospholipid scrambling and phosphatidylserine exposure in apoptotic cells [53–55]. Given that dre-miR-457a mediates the proliferation of TPMs, we speculate that LNC_000668 might regulate phospholipid metabolic process by competing with dre-miR-457a to regulate the expression of XK-related protein, which might play roles on TPMs apoptotic process. However, whether LNC_000668-dre-miR-457a-ENSONIT034160R115166 regulatory network functions during TMPs activation needs experimental verification. In addition, we found that LNC_000792, LNC_000215, LNC_000035 and LNC_000310, which are in *cis* and/or *trans* network, were involved in the ceRNA network as well. In the mRNAs that were targeted by the four lncRNAs, ENSONIT034160R126779 and ENSONIT034160R106484 were the mRNAs that were also the targets for both LNC_000215 and LNC_000035 in the ceRNA network. This highlights the crosstalk of genes in regulatory networks during TPMs activation.

## MATERIALS AND METHODS

### Ethics statement

All experiments were conducted in strict accordance with the Guide for the Care and Use of Laboratory Animals of the National Institutes of Health and Welfare. Sample collection protocol was approved by the Committee on the Ethics of Animal Experiments of the Guangxi Institute of Fisheries (Permit Number: 200099). Animals were humanely sacrificed as necessary to ameliorate suffering.

### Macrophage isolation and *in vitro* stimulation with Hsp70–Sa antigen

*Oreochromis niloticus* brood stocks were obtained from the National Tilapia Seed Farm (Nanning, Guangxi, China). *Streptococcus agalactiae* bacteria cultivation was performed as previously reported [56]. HSP70 protein and Sa antigen was prepared as detailed in our previous study [5]. Peritoneal macrophages were isolated and cultured as in our previous study [5]. The obtained peritoneal macrophages were cultured 1.0×10^6^ cells per well in 24-well plates for 24 h. A final volume of 200 μl macrophages in triplicate were stimulated with 100 μg/ml HSP70, 1.0×10^5^ cells/well Sa antigen, 100 μg/ml HSP70 plus 1.0×10^5^ cells/well Sa antigen and media control at 27°C in 5% CO_2_ for 24 h. Interaction of HSP70 and Sa antigen with macrophages were verified by fluorescence technique as previously reported [5]. Macrophages in all groups were harvested for total RNA isolation.

### Total RNA isolation

Total RNA used for library construction and PCR was isolated from each individual sample using TRIzol reagent (Invitrogen, USA). DNase I was added to remove contaminating genomic DNA. Integrity of RNA was evaluated by using the RNA Nano6000 Assay Kit of the Bionalyzer 2100 system (Agilent Technologies, CA, USA). Purity and quantity of total RNA were tested by using a Nanodrop spectrophotometer (Thermo Scientific, USA).

### Library construction for lncRNA sequencing

We used 3 μg RNA per sample as input material for the RNA preparations. Ribosomal RNA was removed by Epicentre Ribo-zero™ rRNA Removal Kit (Epicentre, USA) followed by rRNA free residue cleaning by ethanol precipitation. Libraries were constructed using the rRNA-removed RNA by NEBNext® Ultra™ Directional RNA Library Prep Kit for Illumina® (NEB, USA) according to the manufacturer's instructions. The library fragments were purified with AMPure XP system (Beckman Coulter, Beverly, USA) to select cDNA fragments between 150 to 200 bp in length. Library quality was evaluated on the Agilent Bioanalyzer 2100 system. The libraries were sequenced on an Illumina Hiseq 2500 platform at the Novogene Bioinformatics corporation (Beijing, China).

### LncRNA data analysis

Firstly, all raw reads with fastq format were processed through perl scripts to obtain the clean data (clean reads) by removing those reads containing adapter or ploy-N and low quality reads. Then, Q20, Q30, and GC content of the clean data were calculated and accessed. Subsequently, the high quality clean reads were mapped with the *Oreochromis niloticus* genome sequence assembly (Bowtie v2.0.6 and Tophat (v2.0.9)). Finally, the mapped clean reads of each sample were assembled by both programs of Cufflinks [57].

### Gene coding potential and its target gene prediction

The CPC, CNCI, and PFAM were used to distinguish mRNAs from lncRNAs. CPC program was used to clarify the coding and non-coding *trans*cripts to evaluate the extent and quality of the ORF by searching the sequences with known protein sequence database [58]. CPC value < 0 was considered as non-coding *trans*cripts. CNCI was used to distinguish coding and non-coding sequences according to the spectrum of the nearby trinucleotide and effectively predict the incomplete and anti-sense *trans*cripts. Pfam Scan (v1.3) was used to identify the occurrence of the known protein family domains in the PFAM database [59]. The *trans*cripts without coding potential were considered as lncRNA candidates. The coding genes 10k/100k upstream and downstream of lncRNA were classified as the *cis* target gene, and the expression level of each *trans*cript was used to identify *trans* target gene of lncRNAs. Depending on the relationships between lncRNAs and their neighboring protein-coding genes, lncRNAs could be classified into six types: (1) sense, (2) anti-sense, (3) bidirectional, (4) intronic, (5) intergenic, and (6) small RNA (sRNA) host lncRNA [28]. In this study, lncRNAs were annotated according to the above classification.

### QPCR

Purified total RNA was synthesized by using the PrimeScript RT reagent (Takara, Japan). QPCR was performed on an ABI 7500 PCR instrument using SYBR green reagents (Life Technologies, Carlsbad, CA) following the manufacturer's protocol. Each qPCR reaction (25 μ L) was comprised of 12.5 μ L 2 × SYBR Green qPCR Master Mix (Life Technologies, Carlsbad, CA), 1 μ L primer, 2 μ L cDNA, and 8.5 μ L H_2_O. The cycling was initiated by a single denaturing cycle of 95°C for 3 min followed by 40 cycles at 95°C for 20 s, 56-60°C for 20 s, and 72°C for 30 s. Specific primers used in this study were listed in Supplementary Table 1. Gene relative expression levels were normalized with β-actin by using 2^−ΔΔCt^ value method [60]. The correlation between the results of RNA-seq and qPCR was calculated using Pearson's correlation method.

### GO and KEGG pathway analyses

The quantification of lncRNAs and coding genes was calculated by Cuffdiff (v2.1.1) [61]. *Trans*cripts with change folds >2.0 and *P* < 0.05 were considered as differentially expressed genes. To explore the roles of these genes, KEGG (http://www.kegg.jp/) was used to confirm the pathway enrichment and GO analysis (http://www.geneontology.org) was performed to identify the roles of differentially expressed lncRNAs and mRNAs. The GO terms included biological process, cellular component, and molecular function. The corresponding target genes of the differentially expressed lncRNAs were mapped to GO terms and integrated discovery DAVID program (http://david.abcc.ncifcrf.gov/). The *p*-value indicated the significance of the pathway, and lower *p*-value indicated higher significance of the pathway.

### Construction of the ceRNA network

LncRNAs or mRNAs can interact with miRNAs through their MicroRNA Recognition Elements (MREs) within a ceRNA network. Firstly, the differentially expressed lncRNAs and/or mRNAs were selected in each sample. To build the ceRNA network, the interactions between differentially expressed lncRNAs and miRNAs were predicted by miRcode program (http://www.mircode.org/), and then lncRNAs and/or mRNAs targeted by miRNAs were analyzed by using TarBase program (http://www.microrna.gr/tarbase). The ceRNA network was built and illustrated by using Cytoscape (v3.4.0). Analyses were performed by Novogene Bioinformatics corporation (Beijing, China).

Statistical analysis Numerical data were analyzed by software GraphPad Prism 5.0 using the two-tailed Student's t-test, ANOVA and the Mann-Whitney test. All data were displayed as the mean± standard error of the mean (SEM). An absolute fold change ≥2, *p*< 0.05 was used to screen differentially expressed lncRNA and mRNA. *p*< 0.05 was considered statistically significant.

### Abbreviations

TPMs: tilapia peritoneal macrophages; Sa: *Streptococcus agalactiae;* TUCP: transcripts of uncertain coding potential; HSP70: heat shock protein 70; ORF: open reading frame; qPCR: quantitative polymerase chain reaction; lncRNA: long non-coding RNA; GO: gene ontology; KEGG: Kyoto Encyclopedia of Genes and Genomes; FPKM: fragments per kilobase of exon per million fragments mapped; MREs: microRNA recognition elements; CeRNA: competing endogenous RNAs.

## SUPPLEMENTARY MATERIALS FIGURES AND TABLES
















