# Supplementary material for: LncRNA and mRNA profiling during activation of tilapia macrophages by HSP70 and Streptococcus agalactiae antigen
Source: Oncotarget. 2017 Sep 30;8(58):98455–70. doi: 10.18632/oncotarget.21427 (PMC5716742; doi:10.18632/oncotarget.21427)
Supplement: Supplementary file 1 [file oncotarget-08-98455-s001.pdf]

LncRNA and mRNA profiling during activation of tilapia macrophages by HSP70 and *Streptococcus agalactiae* antigen

SUPPLEMENTARY MATERIALS

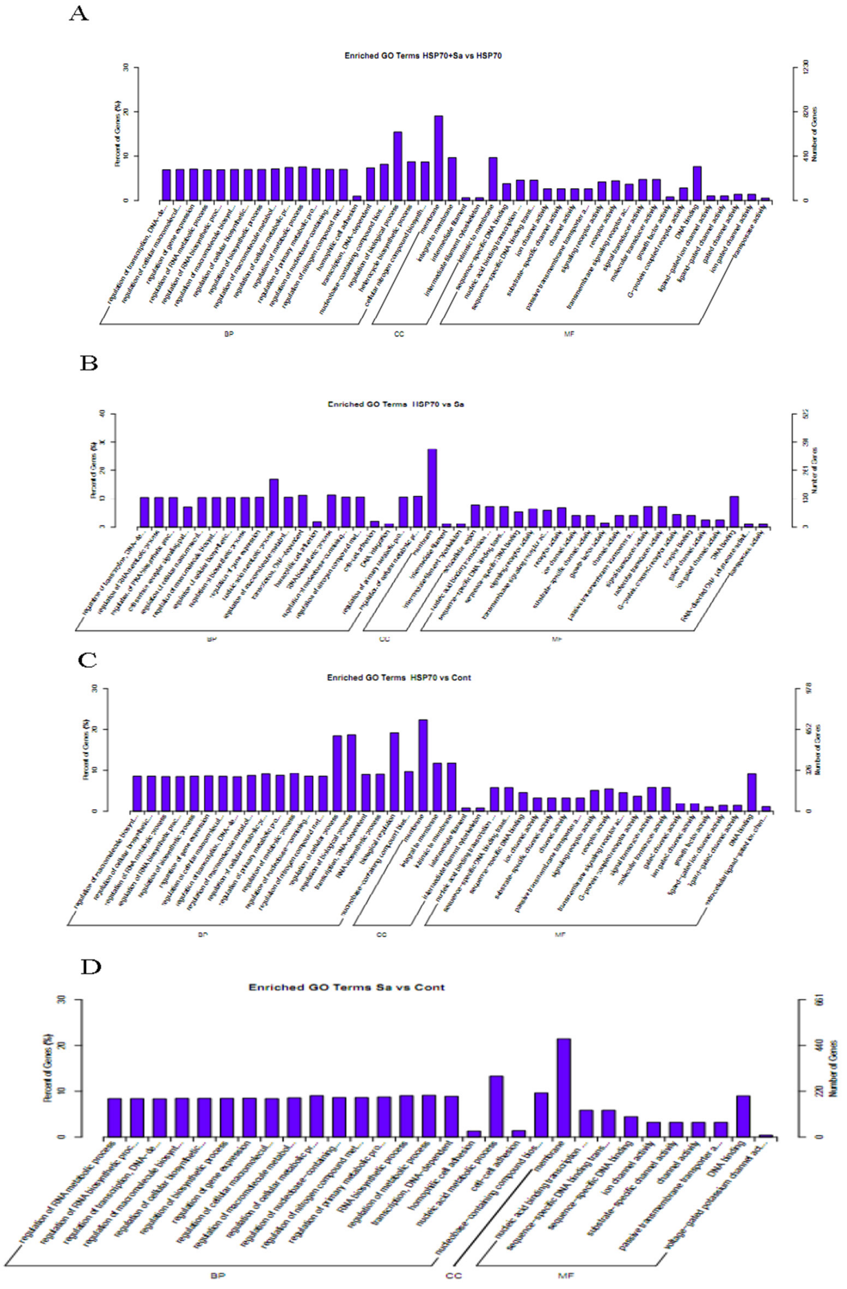

Supplementary Figure 1: GO term enrichment of different expression lncRNAs from four comparison groups. Figures shown the lncRNAs predicted in trans relationship with mRNAs.

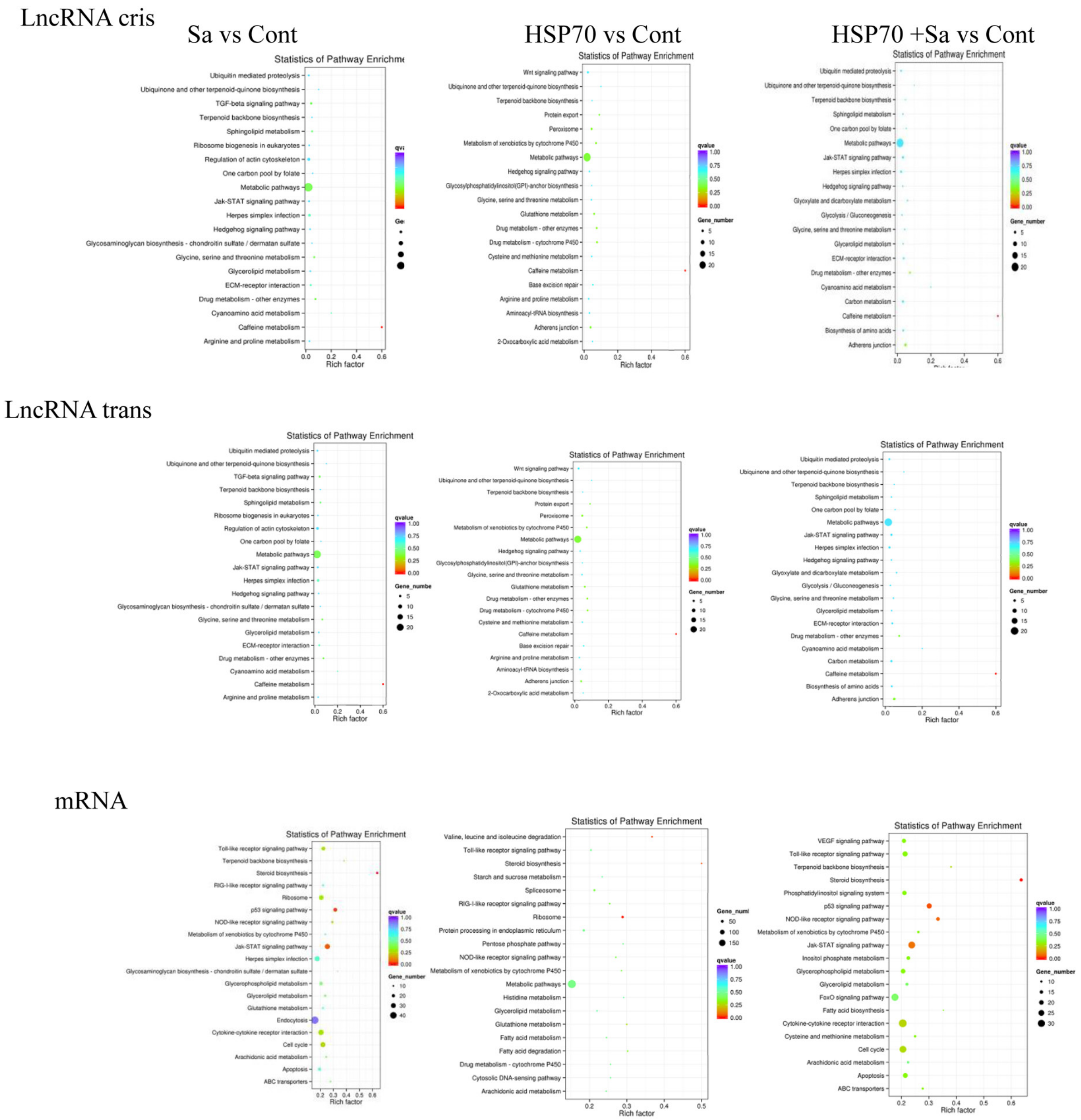

Supplementary Figure 2: KEGG pathways involved in process of tilapia TPMs activation in comparison group Sa vs Cont, HSP70 vs Cont and HSP70 +Sa vs Cont. Figures shown the lncRNAs predicted in cis and trans relationship with mRNAs, respectively.

Supplementary Table 1: Numbers of differentially expressed mRNA, lncRNA and TUCP in each comparison

Differentially expressed gene		HSP70+Sa vs Cont	HSP70+Sa vs Sa	HSP70+Sa vs HSP70	HSP70 vs Cont	Sa vs cont	Total
mRNA	up	977	213	1182	1426	1246	10173
	down	993	217	1962	935	1022	
lncRNA	up	26	12	71	33	36	356
	down	28	20	45	58	27	
TUCP	up	169	33	200	293	233	1782
	down	133	106	366	137	112	
Total		2348	601	3826	2882	2676	

Supplementary Table 1-1: Differentially expressed lncRNA

See Supplementary File 1

Supplementary Table 1-2: Differentially expressed mRNA

See Supplementary File 2

Supplementary Table 1-3: Differentially expressed TUCP

See Supplementary File 3

Supplementary Table 2: Primers used in present study

See Supplementary File 4

Supplementary Table 3: lncRNA and mRNA co-location results

See Supplementary File 5

Supplementary Table 4: lncRNA and mRNA co-expression results

See Supplementary File 6

Supplementary Table 5: Differentially expressen ceRNA network analysis

See Supplementary File 7
