# Supplementary material for: LncRNA and mRNA profiling during activation of tilapia macrophages by HSP70 and Streptococcus agalactiae antigen
Source: Oncotarget. 2017 Sep 30;8(58):98455–70. doi: 10.18632/oncotarget.21427 (PMC5716742; doi:10.18632/oncotarget.21427)
Supplement: Supplementary file 5 [file oncotarget-08-98455-s005.docx]

**Supplementary Table 2**: **Primers used in present study**

| Primer name |  | Primer sequence（5’-3’） | Tm (℃) | Amplicon length（bp） |
| --- | --- | --- | --- | --- |
| beta-actin | F | CCACTTCCTTTTGTCTGGCTTAC | 60 | 267 |
|  | R | CCATTCCGCTTCTACTTCCTG | 58 |  |
| LNC_000002 | F | GCGTGACCAACTACGGAAGC | 60.5 | 292 |
|  | R | TTCGGGAGCAGGCACAATC | 61.5 |  |
| LNC_000035 | F | ACTCAATTTCCGCTCCATCC | 58.9 | 131 |
|  | R | ACCGCCGTTTCTTGCTGTTA | 60.9 |  |
| LNC_000215 | F | TTTTGCTTTGCCTCTACTCAG | 55.5 | 163 |
|  | R | GGCCATAATTTCCTAACCGT | 56.3 |  |
| LNC_000325 | F | TGCGTCGCTGGATCGTT | 58.4 | 167 |
|  | R | AGCTGAGTGAGGGGAGAAGG | 58.2 |  |
| LNC_000358 | F | GGGCAGCATGAGGTTGAGA | 58.8 | 262 |
|  | R | GCAAGCATCAGTCAGCACAGT | 58.5 |  |
| LNC_000360 | F | ATCCGTGGTGATGAATGTTG | 55.9 | 221 |
|  | R | TTCGTTCCTTCTTAGGCTTG | 55.1 |  |
| LNC_000466 | F | TGGTGATTCGTCCATTCG | 548 | 337 |
|  | R | TTCCAGGACATCAGGCAAT | 55.6 |  |
| LNC_000768 | F | CACAGAGGATGAAGCCGACC | 60.1 | 202 |
|  | R | CCTGATAGCCACAGACCGTAGT | 58.6 |  |
| LNC_000792 | F | ACAGACACTTCCTACCCGATTT | 57.3 | 208 |
|  | R | TTCCCAGTCACTTTCAGTTCAA | 57.4 |  |
| LNC_000005 | F | TGGGGAGGGGACTTTTG | 55.6 | 121 |
|  | R | TTATCGTAGAGCGGCAAGAA | 56.5 |  |
| LNC_000310 | F | AGTAGATCAGAGGCATGGGC | 56.5 | 202 |
|  | R | AGAAGCAACAAACTTGGGAAC | 56.3 |  |
| LNC_000354 | F | GAAACCACAGCACCATCG | 54.4 | 83 |
|  | R | TGGGCTTTCTCGTGTATGA | 54.3 |  |
| LNC_000423 | F | CGCATAGACCTCGCCTGAT | 58.4 | 159 |
|  | R | CGAAGCCCGTGTCCAAAT | 58 |  |
| LNC_000424 | F | AGGGAAGTATCAGCAGCATTG | 57.1 | 110 |
|  | R | TGCAGTCATAAAGATCACCCA | 56.3 |  |
| LNC_000428 | F | AGTGAGACGGACATTACAGGG | 56.5 | 103 |
|  | R | CTGAAGTTACATTTGGTGCTGA | 56.7 |  |
| LNC_000449 | F | AAGAGCAAGGTGAGCGACAAG | 59.7 | 499 |
|  | R | CTGAAAGAGTCCATCGCATCG | 60.6 |  |
| LNC_000494 | F | GGGAGGCTGAAGAGGAACAT | 58 | 139 |
|  | R | TGATTCGGACTCTGACAAAACA | 58.1 |  |
| LNC_000521 | F | TTGTTTTGGTGGAACGGTATG | 58.5 | 121 |
|  | R | GCCTGAGGTCTTATGGGTCG | 59.4 |  |
| LNC_000628 | F | GCAAGCTGTTGACAAGAGGAT | 57 | 233 |
|  | R | TGATAGTTCAGGGAGTTGGATG | 56.8 |  |
| LNC_000638 | F | TAGCCCGTGTCTGAAGCAT | 56.2 | 338 |
|  | R | TGGCGGTAATCCACCTCTA | 56.2 |  |
| LNC_000655 | F | AACTTCCGAGCGCAAACT | 55.6 | 80 |
|  | R | TCTCCCGGTCCTACTTCTTC | 55.8 |  |
| LNC_000715 | F | ACTGACAGAAAGTGAGGGAAAG | 55.4 | 235 |
|  | R | GCAGTAATGTAGTGGGAATGTG | 55 |  |
| LNC_000364 | F | CGAACCACGAGACAGAAAGAA | 57.9 | 258 |
|  | R | CGGTATCAGCACATAAGGACG | 58.3 |  |
| LNC_000146 | F | TACTTCGTCAGTCTGTGCTTTT | 54.7 |  |
|  | R | GGTATAGTCCCTTAGCCCTTG | 55.4 |  |
| LNC_000071 | F | CATCCATCGCAAATCTACAA | 54.3 | 224 |
|  | R | TCTATCCCTGCCACGCTA | 54.4 |  |
| LNC_000112 | F | GATGATGATGATGATGATGA | 47.5 | 136 |
|  | R | TTACAGGAAGAGATGAAGT | 43.2 |  |
| LNC_000073 | F | ACCTACACATACCACTGA | 41.1 | 97 |
|  | R | AATACACGCAAGTTAGTCA | 45.8 |  |
| ENSONIG00000000435 | F | TGCGACGAGCCGATGTA | 56.7 | 105 |
|  | R | ACCAACCCACTCACCAAGC | 57.6 |  |
| ENSONIG00000021447 | F | AAGAGGCTGATTGGAAGAAA | 54.2 | 255 |
|  | R | TCGCTGGGAGTCGTTGAA | 57.6 |  |
| ENSONIG00000013416 | F | CTCCTCGTCCTTGCTGCTGT | 60.1 | 305 |
|  | R | AACCGACTTCCCCTTCACC | 58.5 |  |
| ENSONIG00000007093 | F | CGGAGTTTGTGGTGGTTGG | 59.1 | 155 |
|  | R | CGTATGGAAAGAGCGAGCAGTGA | 58.6 |  |
| ENSONIG00000020545 | F | GGATTTTGGGGATTCGG | 55.3 | 150 |
|  | R | TCACCAGGGCTCGCTTT | 56.6 |  |
| ENSONIG00000007635 | F | CCCTTACTCCGTCACTCAAC | 54.4 | 348 |
|  | R | CCATCCACAGACCCAAAAT | 55 |  |
| ENSONIG00000000638 | F | ACAATCTTTCCCTCCATAGCC | 57.6 | 100 |
|  | R | TTACCTCCTTTTCCACCTTCT | 55.2 |  |
| IL-1b | F | TGGACCACCTCAGTTCACC | 55.4 | 255 |
|  | R | GGCTTGTCGTCATCCTTGT | 55.2 |  |
| IL-6 | F | CCGATTGAAGACGGAAGTG | 56 | 279 |
|  | R | AACCGAGTAGATGAGCAGACC | 56 |  |
| IL-10 | F | CACTGCTGCCGTTTCGT | 55.3 | 257 |
|  | R | GTCGTCGTTTGCTTCCTG | 54.1 |  |
| IL-12a | F | AAGCGATTGCGTCTTACCT | 55.1 | 226 |
|  | R | ATAGTGATGGCTCTGATGTGG | 54.7 |  |
| TGFb | F | ATCTTCCGTCTTCAAAACCC | 55.8 | 126 |
|  | R | TGTTCGTATTACCTTGCTGTCA | 56.2 |  |
| TNFa | F | ACAGCCAAGCATCTTTCCG | 58.5 | 279 |
|  | R | TCTCCAGCATTGCCTCCTC | 58.1 |  |
| Arg-1 | F | TCCTGAAGATGCTGGGTGT | 55.4 | 223 |
|  | R | TGTTCGGTGATGTAGACGC | 54.5 |  |
| MHC I | F | GGGATGAAGAAACAGGTGAA | 54.5 | 333 |
|  | R | CCATCTCGGCTCTGTTAGG | 55.4 |  |
| MHC II | F | TCCCTATCCCAACAAAGACG | 57.5 | 381 |
|  | R | AGAAGGTTCCAGCAGCCAC | 57.3 |  |
| CD86 | F | TGTGCTGGGGCTATTCTCA | 57 | 101 |
|  | R | AGGGCAATGTGGTGTTTTCT | 56.9 |  |
| HSP70 | F | AAGAGGCTGATTGGAAGAAA | 54.2 | 257 |
|  | R | TGTCGCTGGGAGTCGTT | 54.3 |  |
| AKT1 | F | GGGTGTTTTCAGAGGAGCG | 57.8 | 208 |
|  | R | GTCCCACAGAAGGTTTTCATAG | 55.9 |  |
| FOXO3a | F | ACAATCTTTCCCTCCATAGCC | 57.6 | 100 |
|  | R | TTACCTCCTTTTCCACCTTCT | 55.2 |  |
| KaT2a | F | TTGGAGGCATCTGTTTTAGG | 55.4 | 312 |
|  | R | GCTCGGTGTAAGGGATTCTA | 54.6 |  |
| HSF-1 | F | TTTTCAGTTCATCTGGCTCG | 56.1 | 100 |
|  | R | CCTGTTCCGCTTTCTTCAC | 55.4 |  |
| PGC-1 | F | ACACTAAGCGGGCAACG | 54.7 | 170 |
|  | R | AGCTGATGATGGAGGAAATG | 54.7 |  |
| TLR-2 | F | TGGGTTTTCTGCTGGAGTAT | 54.7 | 164 |
|  | R | TGTTGTGCCGCAAAGTGT | 55.8 |  |
| TLR-9 | F | TACTGAAGCACCTCTACGGC | 55.5 | 312 |
|  | R | CTCTGGACAGCACGAAAACT | 55.1 |  |
| MyD88 | F | TGCCTTCATCTGCTACTGC | 54.2 | 173 |
|  | R | CCATCCGTTTACACCTCTTC | 54.5 |  |
| NF-κB1 | F | GACAGCAAGGCTCCGAATG | 58.7 | 332 |
|  | R | GGGTGGTAGGTGAAGGGTTT | 57.9 |  |
| PPAR-a | F | GTGGCTGCTATTATCTGCTGT | 54.9 | 129 |
|  | R | GAAGGTGTCATCTGGGTGG | 55 |  |
| PPAR-d | F | CCCATACCTCATCACCTTCG | 57.6 | 183 |
|  | R | ATTCCAGTTTCATCCGCACA | 58.6 |  |
| PPAR-g | F | ATGATGTCGCCTCTGATGAA | 55.8 | 267 |
|  | R | CAGTGAATGAAGCACCGTCT | 55.6 |  |
| Sirt1 | F | AACTCCAATCAGCAAACGC | 55.9 | 159 |
|  | R | TCCCCTTCAGCACAGCAT | 56.4 |  |
| RXR | F | CGGCAGAGGAACAAGGAT | 55.3 | 316 |
|  | R | AGCGATGGGAGAACGATG | 56.1 |  |
| ERK1 | F | CTCTTTTCCGCTTACCCAC | 54.9 | 275 |
|  | R | AGACAAATCACTGACGACCAT | 54.1 |  |
| ERK2 | F | ACCCCTACCTGGAACAATACT | 54.7 | 307 |
|  | R | ACATCGGCAGAGGAGCAT | 55.5 |  |
